# Genetic diversity and population structure of the *Bacillus cereus* group bacteria from diverse marine environments

**DOI:** 10.1038/s41598-017-00817-1

**Published:** 2017-04-06

**Authors:** Yang Liu, Qiliang Lai, Juan Du, Zongze Shao

**Affiliations:** State Key Laboratory Breeding Base of Marine Genetic Resources; Key Laboratory of Marine Genetic Resources, Third Institute of Oceanography, SOA; Collaborative Innovation Center for Exploitation and Utilization of Marine Biological Resources; Key Laboratory of Marine Genetic Resources of Fujian Province, Xiamen, 361005 China

## Abstract

The phylogenetic diversity of marine bacteria belonged to the *Bacillus cereus* group has not been well investigated. Here, we present the genetic diversity and population structure of 71 bacteria from diverse marine environments, using a multilocus sequence typing (MLST) approach and the analyses of digital DNA-DNA hybridization (dDDH) and average nucleotide identity (ANI) based on some representative genomic sequences. The MLST analysis demonstrated that these isolates were highly diverse and a wide distribution in marine environments and some of them showed niche specificity to some extent. They were assigned to 27 sequence types (STs) with 23 novel STs. Phylogenetic analysis of 82 bacteria containing 11 type strains based on MLST discriminated them as 20 clusters including 10 new ones. Both the dDDH and ANI results supported the proposition that each of 20 clusters represented one independent species, including 10 putative novel species. Values of 98.3% of MLST similarity and 96.2% of ANI were proposed as the standard for the species definition of this group. In summary, the first insight into the phylogenetic diversity of the group bacteria from marine environments will contribute to better understanding of their ecological role and evolution in contrast with terrestrial environments.

## Introduction

*Bacillus cereus* group comprises 11 closely related species, including the first described species *B. anthracis*, along with *B. cereus*, *B. thuringiensis*, *B. mycoides*, *B. weihenstephanensis*, *B. pseudomycoides* and the recently identified “*B. gaemokensis*”, “*B. manliponensis*”, *B. cytotoxicus*, *B. toyonensis*, “*B. bingmayongensis*” (Three species “*B. gaemokensis*”, “*B. manliponensis*” and “*B. bingmayongensis*” are effectively but not yet validly published and thus are in quotation marks throughout in this study)^1^. In comparison with their tremendous contributions for production of numerous enzymes^2^ and metabolites^3^, removal of various heavy metals^4^ and persistent organic pollutants^5^ and growth promotion of animals and plants as probiotics^6^, much attention has been focused on the epidemiology and pathogenesis of the *B. cereus* group bacteria^7^. More specifically, *B. anthracis*, the etiologic agent of anthrax, can result in high mortality for human and ungulate herbivore and was even used as a biological weapon in a terrorist attack^8^. *B. cereus* as an opportunistic pathogen is responsible for food poisoning caused by a range of protein toxins, characterized by either nausea and vomiting or abdominal pain and diarrhea as well as a wide range of serious invasive infections^9^. Occasionally, the presence of toxin genes is determined using a variety of methods in *B. mycoides*, *B. weihenstephanensis*, *B. cytotoxicus* and other^10^. *B. thuringiensis* is an entomopathogenic bacterium largely attributed to insecticidal crystal proteins encoded by the *cry* genes, which generally locate on plasmids^11^.

The taxonomy of the *B. cereus* group strains is the cornerstone for better understanding of phylogenetic relationships and population diversity. In the past several decades, bacterial identification and taxonomic classification of this group have been extensively investigated by using traditional phenotypes (e.g., morphology, physiology, biochemical characteristics, etc.) and genotypes (e.g., ribotyping, random amplified polymorphim DNA, multilocus sequence typing (MLST), etc.)^12–16^. Given the simplicity, reproducibility and discrimination among these approaches, MLST is an outstanding method for elucidating clonal relationships of bacteria within this group. Meanwhile, with the advent of the genomic era, a large number of publicly available genomic sequences provide a promising avenue for bacterial species delineation using digital DNA-DNA hybridization (dDDH) and average nucleotide identity (ANI). Recently, a number of studies combining with dDDH and ANI have been successfully applied to some genera and species^1, 17, 18^. However, this approach has not yet been applied to the *B. cereus* group.

The bacteria of the *B. cereus* group occupy a wide range of habitats, ranging from soil to water and from plants and animals to food, probably owing to the metabolic diversity endowing them with ecological adaptation of different environments and strong survivability of spores allowing them to better withstand hostile conditions and to better disperse^19, 20^. Their population structure and phylogenetic diversity in freshwater and terrestrial environments has been extensively investigated in recent years using several MLST schemes^14–16^. However, to the best of our knowledge, they are less well understood in diverse marine environments, particularly in the deep sea. In last decades, a large number of bacteria within this group were recovered from various marine samples after enrichment with heavy metals, crude oil and the polycyclic aromatic hydrocarbons under various environmental conditions like low temperature and high salinity. These samples used were collected globally, mainly seawater of different layers and sediments from both coastal and pelagic areas, from the Pacific Ocean^21, 22^, the Indian Ocean^23^, the South China Sea^24^, etc., awaiting further analyses on their phylogenetic and ecological diversity.

The aim of this study is to investigate the diversity and niche specificity of bacteria affiliated to the *B. cereus* group in marine environments by means of an MLST scheme, and to further elucidate their taxonomic position by measurements of dDDH and ANI, using 71 isolated strains together with 11 previously published type strains (Table 1).

**Table 1:** Complete list of 82 strains used in this study with detailed annotations.

NO.	MCCC NO./Names	Cluster	Samples	Areas
BC01	1A00235	01	Sediment	The Pacific Ocean
BC02	1A00239	01	Sediment	The Pacific Ocean
BC03	1A00262	01	Sediment	The Pacific Ocean
BC04	1A00265	01	Sediment	The Pacific Ocean
BC05	1A00266	01	Sediment	The Pacific Ocean
BC06	1A00267	01	Sediment	The Pacific Ocean
BC07	1A00268	01	Sediment	The Pacific Ocean
BC08	1A00274	01	Sediment	The Pacific Ocean
BC09	1A00298	11	Sediment	The Pacific Ocean
BC10	1A00359	08	Sediment	The Pacific Ocean
BC11	1A00360	11	Sediment	The Pacific Ocean
BC12	1A00361	10	Sediment	The South China Sea
BC13	1A00365	15	Sediment	The Pacific Ocean
BC14	1A00394	01	Sediment	The Pacific Ocean
BC15	1A00395	01	Sediment	The Pacific Ocean
BC16	1A00418	11	Sediment	The Pacific Ocean
BC17	1A00419	01	Sediment	The Pacific Ocean
BC18	1A00430	01	Sediment	The Pacific Ocean
BC19	1A00431	01	Sediment	The Pacific Ocean
BC20	1A00432	11	Sediment	The Pacific Ocean
BC21	1A00447	01	Sediment	The Pacific Ocean
BC22	1A00460	01	Sediment	The Pacific Ocean
BC23	1A00495	11	Sediment	The Pacific Ocean
BC24	1A00594	06	Sediment	The Pacific Ocean
BC25	1A00732	14	Sediment	The Pacific Ocean
BC26	1A00841	10	Sediment	The Pacific Ocean
BC27	1A01056	01	Seawater	The Indian Ocean
BC28	1A01400	11	Sediment	The Indian Ocean
BC29	1A01404	11	Sediment	The South China Sea
BC30	1A01406	03	Sediment	The South China Sea
BC31	1A01412	02	Sediment	The South China Sea
BC32	1A01414	03	Sediment	The South China Sea
BC33	1A01874	11	Sediment	The Pacific Ocean
BC34	1A02143	03	Sediment	The South China Sea
BC35	1A02146	05	Sediment	The South China Sea
BC36	1A02161	02	Sediment	The South China Sea
BC37	1A04040	10	Sediment	The South China Sea
BC38	1A04083	11	Seawater	The Pacific Ocean
BC39	1A04098	13	Sediment	The South China Sea
BC40	1A05675	15	Sediment	The South China Sea
BC41	1A05688	09	Sediment	The South China Sea
BC42	1A05689	10	Sediment	The South China Sea
BC43	1A05691	10	Sediment	The South China Sea
BC44	1A05942	07	Sediment	The Indian Ocean
BC45	1A06182	04	Seawater	The Pacific Ocean
BC46	1A06183	11	Seawater	The Pacific Ocean
BC47	1A06184	11	Seawater	The Pacific Ocean
BC48	1A06185	11	Seawater	The Pacific Ocean
BC49	1A06187	04	Seawater	The Pacific Ocean
BC50	1A06188	04	Sediment	The Pacific Ocean
BC51	1A06189	04	Seawater	The Pacific Ocean
BC52	1A06191	04	Sediment	The Pacific Ocean
BC53	1A06192	04	Seawater	The Pacific Ocean
BC54	1A06193	04	Sediment	The Pacific Ocean
BC55	1A06194	04	Seawater	The Pacific Ocean
BC56	1A06359	01	Sediment	The Gulf of Mexico
BC57	1A06360	01	Sediment	The Gulf of Mexico
BC58	1A06361	01	Sediment	The Gulf of Mexico
BC59	1A06374	15	Sediment	The Gulf of Mexico
BC60	1A06375	15	Sediment	The Gulf of Mexico
BC61	1A06376	15	Sediment	The Gulf of Mexico
BC62	1A06378	15	Sediment	The Gulf of Mexico
BC63	1A06928	04	Seawater	The Pacific Ocean
BC64	1A06935	11	Seawater	The Pacific Ocean
BC65	1A06937	04	Seawater	The Pacific Ocean
BC66	1A06939	04	Seawater	The Pacific Ocean
BC67	1A06940	04	Seawater	The Pacific Ocean
BC68	1A06941	04	Seawater	The Pacific Ocean
BC69	1A06942	04	Seawater	The Pacific Ocean
BC70	1A08292	11	Sediment	The Arctic Ocean
BC71	1A08490	11	Sediment	The Arctic Ocean
BC72	*B. anthracis* ATCC 14578^T^	02	Other (Cattle)	Terrestrial Environments
BC73	*B. cereus* ATCC 14579^T^	11	Other (Air, farmhouse)	Terrestrial Environments
BC74	*B. cytotoxicus* NVH 391–98^T^	19	Other (Food)	Terrestrial Environments
BC75	*B. mycoides* DSM 2048^T^	12	Soil	Terrestrial Environments
BC76	*B. pseudomycoides* DSM 12442^T^	17	Soil	Terrestrial Environments
BC77	*B. thuringiensis* ATCC 10792^T^	10	Other (Insect)	Terrestrial Environments
BC78	*B. weihenstephanensis* DSM 11821^T^	12	Other (Food)	Terrestrial Environments
BC79	*B. toyonensis* BCT-7112^T^	09	Soil	Terrestrial Environments
BC80	“*B. gaemokensis*” BL3–6^T^	16	Sediment	The Yellow Sea
BC81	“*B. manliponensis*” BL4–6^T^	20	Sediment	The Yellow Sea
BC82	“*B. bingmayongensis*” FJAT-13831^T^	18	Soil	Terrestrial Environments

## Results

### Sequence diversity

The MLST sequences for the P scheme^16^ were from 348 bp (*purH*) to 504 bp (*gmk*) in length, and seven concatenated sequences produced a 2,829-bp fragment (Table 2). Among the 82 strains, the number of alleles for per gene varied from 28 to 34, and the number of polymorphic sites (S) ranged from 82 to 191 (Table 2). The gene *ilvD* possessed the highest number of alleles (34), and correspondingly the highest number of polymorphic sites (191), whereas *gmk* and *tpi* possessed the lowest number of alleles (28) and polymorphic sites (82). No insertions or deletions were observed in any sequences except for type strain *B. manliponensis* BL4–6^T^ with a six-base insertion in *ilvD* gene. The nucleotide diversity (*π*), which is defined as the average number of nucleotide differences per site between two randomly selected sequences, varied from 0.0199 (*tpi*) to 0.0856 (*ilvD*). Seven genes exhibited mean G + C content from 38.0 to 44.8 mol%, which were higher than those of the genomic sequences (ca. 35 mol%) of the *B. cereus* group bacteria from the GenBank database. A considerable variation in the *Ka*/*Ks* ratio was observed in Table 2, suggesting that *tpi* and *glpF* were under higher selective pressure than *gmk*, *ilvD*, *pta*, *purH* and *pycA*. However, all values were far below one, indicating that these genes were most likely selectively neutral and thereby suitable for MLST analysis. Consistently, the Tajima’s D values, which measures deviation from the standard neutral model of evolution, ranged from −1.8967 to −0.7817 (Table 2). The evolution of these genes was likely driven by neutral selection, which is typical for housekeeping genes.

**Table 2:** Characteristics of seven housekeeping genes from 82 strains.

Gene	Size (bp)	No. of alleles	S	*π*	G + C	*Ka*/*Ks*	D
*glpF*	372	33	95	0.0302	38.4	0.0745	−1.8967
*gmk*	504	28	157	0.0434	38.0	0.0242	−1.4757
*ilvD*	393	34	191	0.0856	44.8	0.0150	−1.4036
*pta*	414	32	89	0.0247	40.8	0.0173	−1.5578
*purH*	348	31	148	0.0560	38.7	0.0246	−1.6714
*pycA*	363	29	114	0.0665	40.2	0.0238	−0.7817
*tpi*	435	31	82	0.0199	44.1	0.1165	−1.6889

Notes: S, number of variable sites; *π*, nucleotide diversity (per site); G + C, the mean G + C content; *Ka*/*Ks*, the number of non-synonymous substitutions per non-synonymous site/the number of synonymous substitutions per synonymous site; D, Tajima’s D value.

The Index of Association (*I*_A_) was calculated to estimate the degree of association and recombination between alleles at different loci based on the allelic profile data^25^. Values of both the classical and standardized Index of Association (*I*_A_ = 5.5542 and ${I}_{{\rm{A}}}^{{\rm{S}}}$ = 0.9256, respectively) were significantly different from zero, and the pairwise variance (V_D_ = 4.3022) was greater than the 95% critical value (L = 0.6901) when all isolates were included in the analyses (P < 0.001), indicating that there was a high level of linkage disequilibrium. Therefore, the result demonstrated that there were limited recombinational events and a clonal structure within the *B. cereus* group.

### STs, CCs, distribution and population of STs

**Figure 1:**
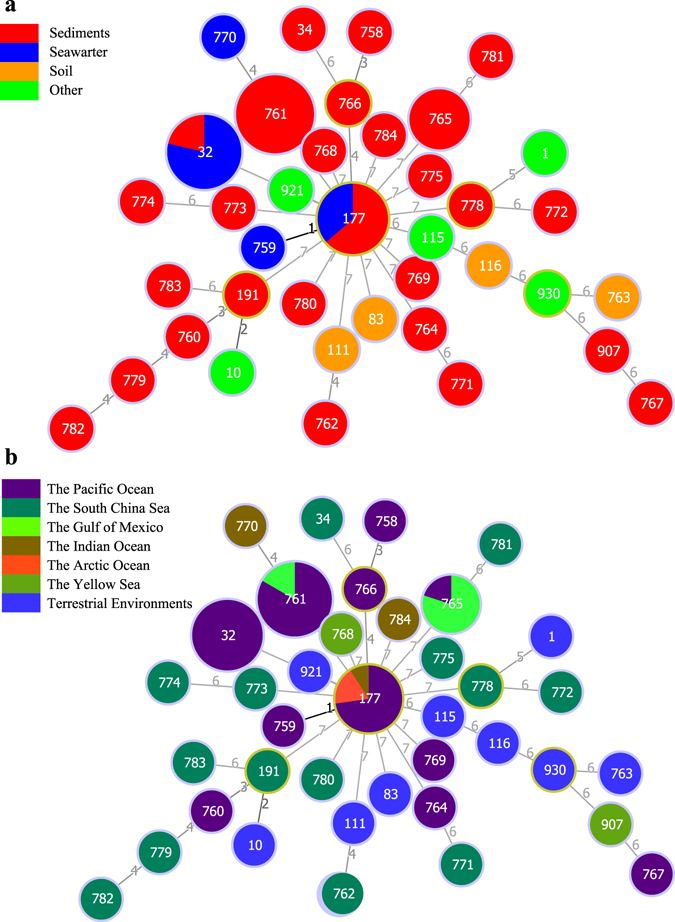
Minimum spanning tree reflecting clonal relationships of sequence types (STs) for 82 *B. cereus* group bacteria constructed using goeBURST. Each ST is represented by a circle, and the size of the circle is logarithmically proportional to the number of strains represented by the ST. Exact numbers of isolates per ST are given in Supplementary Table S1. The numbers connecting the circles indicate the number of genes of difference genes between isolates. (**a**) The isolates are colored according to isolated samples, as given in the key at upper left. (**b**) The isolates are colored according to isolated areas, as given in the key at middle left.

The seven loci of the 71 marine strains (27 STs) and 11 type strains (11 STs) yielded 38 unique sequence types (STs), of which 23 STs were first discovered and deposited in the MLST database (Supplementary Table S1). The most frequent type, ST 761, was shared by 18 strains, followed by the other two types, ST 32 and ST 117, which were shared by 14 and 11 isolates, respectively. The type ST 756 was found in five strains. The other 34 types were represented by only one strain. Thirty-eight STs contained one clonal complexes (CC) and 36 singletons at the single locus variant level by the Global Optimal eBURST (goeBURST)^26^. The sole CC was formed by ST 177 and ST 759, which contained 12 strains (Fig. 1). However, the evolutionary relationship among the 36 singletons could not be inferred based on STs, neither between these singletons and the defined CC doublet.

Further, we analyzed the distribution of STs for 71 strains and two type strains from marine environments. In spite of exhibition of STs for nine type strains in Fig. 1, they were not included in the distribution analyses according to their terrestrial origin. As illustrated in Fig. 1a, ST 761 (18 strains) and ST 765 (5 strains) distributed only in sediment samples. In contrast, ST 32 (14 strains) and ST 177 (11 strains) distributed in both sediment and seawater samples. In sediment samples, the occurrence frequency of STs was 50%, significantly higher than that in seawater (23.5%). Similarly, we also analyzed the distribution of STs in different oceans or marine areas (Fig. 1b). ST 32 representing 14 strains was only found in the Pacific Ocean. On the contrary, ST 177 distributed more widely and was found in the Pacific Ocean, the Indian Ocean, and the Arctic Ocean. Occurrence frequency of STs in the South China Sea (each of the 14 strains represents a unique ST) was considerably higher than those of STs in other marine areas; for example, in 45 strains from the Pacific Ocean, 12 STs were retrieved.

**Figure 2:**
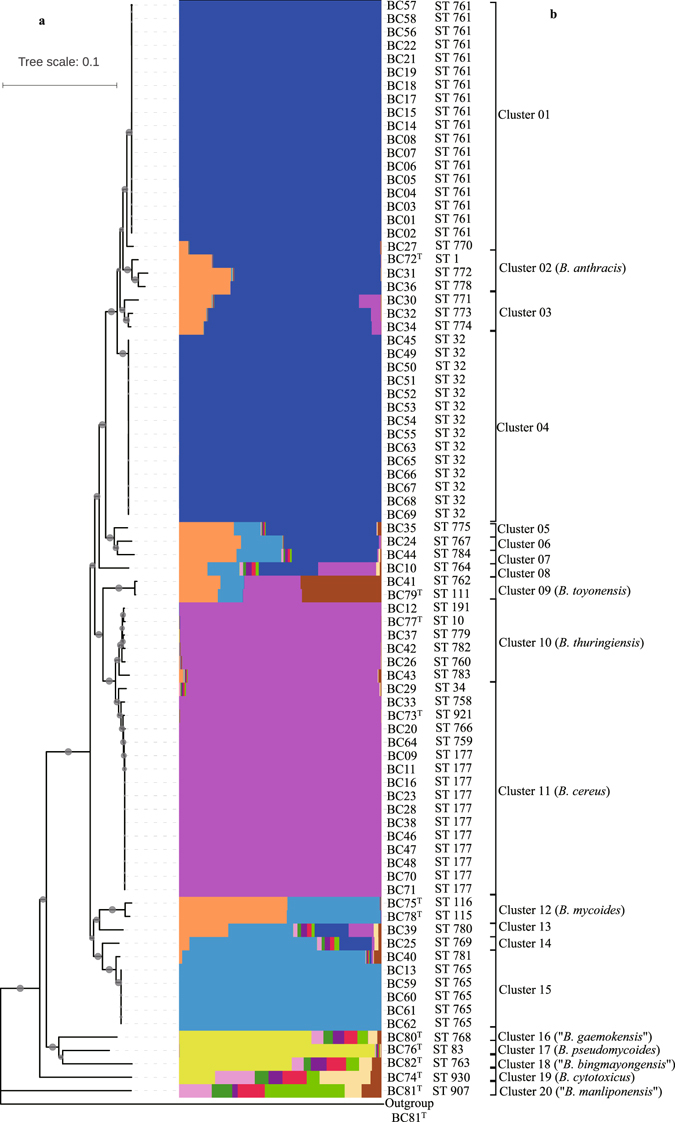
Diagrams denoting population structure and phylogeny of 82 bacteria of the *B. cereus* group. (**a**) The neighbor-joining phylogenetic tree of this group of strains was constructed on the basis of seven concatenated sequences using the software MEGA version 5.05. Bootstrap values (expressed by gray circles with different diameters) are shown at branch points. Bar, 0.1 nucleotide substitution rate (*K*_nuc_) units. *Bacillus subtilis* ATCC 6051^T^ was used as an outgroup. (**b**) Proportions of ancestral subpopulations of all strains and different colors representing distinct assumed subpopulations corresponding to Cluster 01 to Cluster 20.

All STs for 82 bacteria of the *B. cereus* group were analyzed using Structure software^27^. Multiple runs with K values from 2 to 20 showed maximal posterior probability at K = 12, suggesting that the collected the *B. cereus* group strains were descendants of 12 distinct ancestor populations. The rebuilding of population structure showed all STs were attributed to 20 distinct subpopulations, which corresponded to 20 clusters/species described below. There is little admixture of ancestral sources among these subpopulations, such as clusters 1, 4, 10, 11 and 15 (Fig. 2a); thus the STs within each subpopulation were highly homogenous. On the contrary, an obvious admixture of ancestral sources occurs within the subpopulations, such as cluster 5–8 and 16–20 (Fig. 2a).

### Phylogenetic analysis

Generally, the phylogenetic tree of a single housekeeping gene is consistent with that of MLST, but the latter provided a better resolution and presented a robust topology with high bootstrap values. Therefore, a neighbor-joining (NJ)^28^ tree based on seven concatenated housekeeping genes was generated using the software MEGA version 5.05^29^. As shown in Fig. 2b, the MLST tree revealed a high genetic diversity; the 82 strains were divided into 20 clusters, labelled Cluster 01 to Cluster 20. Each cluster represents a unique species based on the following dDDH/ANI analyses. The assignment of 82 strains to each cluster thoroughly described in Table 1.

In more details, Cluster 01 was the largest group, including 19 strains, and had two branches represented by ST 761 and ST 770. Cluster 02 represented *B. anthracis*, and accommodated type strain ATCC 14578^T^ and two strains from the sediment of the South China Sea. Cluster 03 included three strains with unique STs, corresponding to ST 771, ST 773 and ST 774. The third largest group, Cluster 04, contained 14 strains, all from the seawater of the Pacific Ocean, and displayed the same ST (ST 32).

Cluster 09 attributed to the species *B. toyonensis*, including type strain BCT-7112^T^ (ST 111) and strain BC41 (ST 762). Cluster 10 included five marine bacteria and type strain *B. thuringiensis* ATCC 10792^T^, each owns a unique ST, while these types were closely related to each other. Cluster 11, the second largest group, contained 15 marine strains and type strain *B. cereus* ATCC 14579^T^ and was represented by 6 STs in total. Interestingly, in this cluster, 11 isolates of ST 177 were distributed widely, with nine strains from the Pacific Ocean and two from the Arctic Ocean. Cluster 12 harbored two previously established species, represented by type strains *B. mycoides* DSM 2048^T^ and *B. weihenstephanensis* DSM 11821^T^. However, our results indicate that they should be conspecific. Cluster 15 was split into three branches corresponding to 3 STs, represented by 6 strains. Other 11 clusters were each represented by a single strain and corresponded to a unique ST. Thus, in addition to 10 previously established species, 10 new taxa were demonstrated in marine environments.

### The dDDH and ANI analyses

For further validation of the phylogeny based on MLST, the dDDH and ANI analyses were carried out in this study. To ensure at least one strain representative for each cluster from Cluster 01 to 20, 21 genomic sequences of our isolates were determined. The 11 of reference bacteria were obtained from the GenBank database. Therefore, a total of 32 bacterial genomes were analyzed (Supplementary Table S2). The dDDH and ANI values between pairwise strains were shown in Supplementary Table S3. Considering 70% DDH values as the gold standard for the species boundary in bacterial taxonomy^30^, 32 strains were classified into 20 species, perfectly corresponding to the 20 clusters of the MLST analysis. However, the dDDH values between *B. cereus* bacteria and *B. thuringiensis* bacteria were slightly but not significantly above 70%, indicating once again that the two species were of the closest relationship. Furthermore, ANI analysis presented the same phylogeny as obtained by dDDH analysis. According to 95–96% ANI criteria for species definition^31^, 32 strains were also divided into 20 species, corresponding to the same 20 clusters. Therefore, among 20 clusters, 10 belonged to the well-defined species, each of which was represented by a type strain; while the remaining 10 clusters each represented a putative novel species.

**Figure 3:**
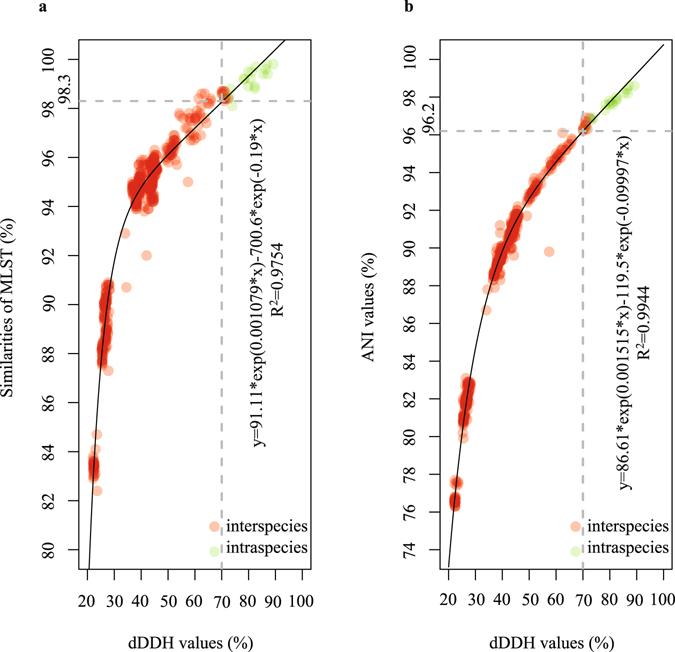
Correlation analyses between the dDDH values and MLST similarities, dDDH and ANI values for 32 strains within this group. The vertical line indicates a 70% dDDH threshold. (**a**) The horizontal line (y = 98.3) indicates the estimated MLST similarity threshold (inter-species) corresponding to 70% dDDH threshold, as given in the key at left. (**b**) The horizontal line (y = 96.2) indicates the estimated ANI threshold (inter-species) corresponding to 70% dDDH threshold, as given in the key at right.

The correlation between the dDDH values and MLST similarities, dDDH and ANI values for 32 strains were determined by a nonlinear simulate analysis method, respectively. The dDDH values were highly correlated with the MLST similarities (R^2^ = 0.9754). Based on the simulative logarithmic equation of y = 91.11*exp(0.001079*x) − 700.6*exp(−0.19*x), 70% DDH was equivalent to a 98.3% MLST similarity (Fig. 3a). The dDDH values were also strongly correlated with the ANI values (R^2^ = 0.9944). Based on the simulative exponential equation (y = 86.61*exp(0.001515*x) − 119.5*exp(−0.09997*x)), 70% dDDH corresponded to a 96.2% ANI (Fig. 3b). The correlation analyses described above indicated that both 98.3% of MLST similarity and 96.2% of ANI could be used as the species threshold for bacteria of the *B. cereus* group.

## Discussion

Bacteria of the *B. cereus* group are the subject of growing interest because of their considerable significance in agriculture and industry, especially in the area of food safety and health of human and animals^7^. They are ubiquitously found in diverse environments and have been isolated from a wide variety of niches for hundreds of years, including the multiple organs of plants, different kinds of human and animals, water from ponds, rivers and lakes, soils of different texture and regions, various types of food and other locations (to get more detailed information of isolation origins for bacteria within the *B. cereus* group, please refer to http://mlstoslo.uio.no/). However, their phylogenetic diversity in marine environments has not been determined to date, although a large number of bacteria have been isolated from various marine environments in our laboratory and other research teams. In this report, we applied MLST, dDDH and ANI analyses to explore their genetic diversity and geographic distribution of this group.

In this study, we found that the 71 marine isolates contained 27 STs that consisted of 23 new STs and four previously identified STs. These isolates were not only widely distributed in marine environments, but also extraordinarily rich in diversity and contained numerous novel genotypes. The marine environment is complex and unique compared with terrestrial environments, characterized by high salinity, oligotrophy and weak alkalinity (pH 7.9–8.4)^32^ in general and other properties in special niches, which certainly contribute to the development of the genetic, physiological and metabolic diversity of marine bacteria.

In addition to the new phylotypes, it is also noteworthy that some marine strains belonged to *B. anthracis*, *B. cereus* and *B. thuringiensis* species. Two strains, designated BC31 and BC36, were affiliated with *B. anthracis* species based on dDDH and ANI analyses. In consideration of their phylogenetic position and the pXO1-like plasmid of BC36 (unpublished data), two strains differed from traditionally described *B. anthracis*, and therefore should be allocated to anomalous *B. anthracis*. In general, *B. cereus* is a ubiquitous opportunistic pathogen and can result in the food poisoning when ingested by humans. Among the 71 marine strains, 14 belonged to *B. cereus* species. Furthermore, the enterotoxin genes (*hblCDA*, *nheABC*, *cytK* and *entFM*)^33^ were detected in most of the genomic sequences of 32 strains using a local BLASTN method (unpublished data), indicating that they have a relatively wide distribution in bacteria of this group, not just limited to *B. cereus*. Accordingly, the bacteria of this group from marine environments, including *B. cereus*, should be given the same attentions as bacteria from terrestrial environments. Four bacteria belonged to *B. thuringiensis*, but did not contain *cry* genes that encode insecticidal crystal proteins (Cry). This is in agreement with our previous report that the presence or absence of *cry* genes cannot be used as a phenotypic characteristic for discriminating *B. thuringiensis* from other^1^.

Evidently, the abundance of bacteria in sediment samples is significantly higher than that in seawater samples, and they possess more diverse STs. This is in accordance with many previous studies^34–36^. Compared with the water column, sediments retain more organic materials that are resistant to degradation, are less proteinaceous and other fragile materials^37^. Sediment thus more closely resembles the soil environment, where *Bacillus* bacteria thrive. Likewise, occurrence frequencies of novel STs were much higher in the South China Sea than in other areas. We speculated that two reasons may account for this phenomenon. On the one hand, the indigenous bacteria of this group isolated from the South China Sea probably possessed more rapidly diversification rates relative to them in other areas because they enjoyed the large input of nutrients from coastal areas. On the other hand, the bacteria isolated from the South China Sea, might have originated from terrestrial environments by watercourse and/or anthropogenic activities, were forced to modify the phenotypic and genotypic characterizations in order to adapt harsh ocean environments, that is, stress-driven evolution. No matter what kind of evolution was driven, they resulted in a much richer diversity of *B. cereus* group isolates in the South China Sea compared with other areas. Certainly, we cannot also completely rule out the possibilities of a lower sampling or less effective cultivability in marine environments than other from the South China Sea. The different assumptions would need to be further confirmed by comparative genomics analysis and other in the following studies.

The classification and taxonomic separation of the *B. cereus* group strains is the cornerstone for a better understanding of their phylogenetic relationships and population structure^38^. In spite of this, it has long been a controversial topic for bacteriologists in distinguishing these species within this group^39^.

Traditionally, these organisms of this group have been differentiated on the basis of diverse phenotypic characteristics, in particular pathogenic potential^7^. For example, *B. anthracis* is the etiological agent of anthrax and an obligate pathogen threatening to human and animal health^8^. *B. cereus* is an opportunistic pathogen and often associated with human food poisoning^10^. *B. thuringiensis* has long been regarded as an insect pathogen owing to the formation of intracellular protein crystals during sporulation^11^. But, given that the phenotypic characteristics depend on environmental conditions, such as media, temperature, light, etc., some strains varied in reproducibility of phenotypes. In such case, the current practices in phenotypic typing for the *B. cereus* bacteria have been questioned.

Genotypic typing approaches on the basis of 16S rRNA gene and single housekeeping genes can overcome the shortcomings of phenotypic analyses, and therefore have been widely used in many studies^40–42^. But, the previous studies were unable to provide an accurate and consistent classification for the phylogenetically interspersed species within this group probably because of the high conservation, horizontal gene transfer and recombination of rRNA and housekeeping gene. For example, the 16S rRNA gene can identify a bacterium to the *B. cereus* group, but cannot assign it accurately to a certain species because of its low discrimination^1^. In our previous study, a housekeeping gene *pycA* was capable of rapidly distinguishing almost all close relatives of this group^1^, which has proven useful for the present study (see Supplementary Fig. S1b). However, as shown in Supplementary Fig. S1b, the use of a shortened *pycA* gene sequence (363 bp) provides inadequate genetic information to describe the accurately phylogenetic relationships of bacteria within this group relative to the MLST analysis^1^ (see Supplementary Fig. S1a). The taxonomic affiliations of bacteria of this group might be wrongly attributed because of horizontal gene transfer of a single housekeeping gene^43^. For example, two strains, *Bacillus cereus* AH187 and *Bacillus cereus* G9241, belonged to two different species on the basis of 64.2% of dDDH and 95.7% of ANI. However, these two strains affiliated to the same species based on a 98.2% similarity of *gyrB* gene. In 2015, the taxonomic status of the bacteria within the *B. cereus* group was clearly established using whole-genome sequences by our group in cooperation with German colleagues^1^. However, species discrimination based on whole-genome sequencing is rather expensive even today.

In view of the aforementioned situation, MLST was applied to investigate the phylogenetic relationship of marine bacteria of the *B. cereus* group in this study. These strains can be divided into 20 clusters corresponding to 20 species that were further confirmed using the dDDH and ANI analyses based on genomic sequences. Among 10 new clusters, six overlapped with those found in our previous report: Cluster 01 in this study corresponded to Group BCG 12, Cluster 03 corresponded to BCG 14, Cluster 04 corresponded to BCG 10, Cluster 06 corresponded to BCG 22, Cluster 13 corresponded to BCG 21, and Cluster 14 corresponded to BCG 11^1^. The other four clusters did not correspond with any clusters from our previous study^1^. Compared with the studies of Priest *et al*.^16^, Barker *et al*.^44^, Hoffmaster *et al*.^45^ and Didelot *et al*.^46^, the analyzed isolates were grouped into three clades including eight lineages with the exception of the “Others” lineage. The closely related *B. cereus* group isolates were roughly clustered with three major clades in the studies of Kim *et al*.^47^ and Cardazzo *et al*.^48^. Although these findings were nearly the same for the *B. cereus* group isolates, these clades and lineages were arbitrarily defined, taking into consideration the relationships between isolates in multiple phylogenetic trees. Moreover, these clades and lineages were also not further validated using other approaches. Consequently, we recommend that the taxonomic and phylogenetic analysis of the *B. cereus* group bacteria should combine a variety of methods in future studies.

The correlation analyses between the dDDH values and MLST similarities, dDDH and ANI values provided two helpful thresholds for the species definition of the *B. cereus* group bacteria. In general, an ANI value of approximately 95–96% was used as the cut-off for species demarcation^31^. In our analysis, this threshold value was slightly raised to 96.2% which will provide more accurate taxonomic relationships of the closely related strains within the *B. cereus* group. Up to the time of writing this manuscript, the MLST database for the *B. cereus* group strains contained 1,272 STs among 1,547 isolates corresponding to the P scheme^16^. Based on the 98.3% MLST similarity, the precise and detailed classification of 1,547 isolates should be feasible in a future study.

In summary, our report uncovers for the first time the genetic diversity of the *B. cereus* group bacteria from diverse marine environments, based on MLST, dDDH and ANI analyses. These isolates are highly diverse with a wide distribution in marine environments and harbor many novel STs, including 20 clusters. Threshold values (98.3% of MLST similarity and 96.2% of ANI) are proposed as the standard for the species definition of this group. The results of this study are beneficial for understanding the adaptation and diversification of the *B. cereus* group to diverse marine environments.

## Methods

### Bacterial isolates and DNA extraction

Seventy-one strains of the *B. cereus* group from the Marine Culture Collection of China (MCCC) were included in this study (Table 1). Specifically for isolated habitats, 54 of them were isolated from the sediments and 17 from the seawater. Among these strains, 50 were from the Pacific Ocean, 9 from the South China Sea, 7 from the Gulf of Mexico, 3 from the Indian Ocean and 2 from the Arctic Ocean. Moreover, all type strains excluding type strain *B. anthracis* ATCC 14578^T^ from multiple Microbiological Culture Collection Center abroad have currently preserved in the MCCC, and have been used as reference strains (Table 1). For the original source of type strains, four were from soil samples, two from the sediments, and five from other, including cattle, air, food and insect. Apart from two type strains from the Yellow sea, nine were from the terrestrial habitats (Table 1). All strains were incubated on modified Luria-Bertani medium with 3% NaCl (w/v) at pH 7.0 and 32 °C. Genomic DNA was extracted using the SBS extraction kit (Shanghai SBS Genetech Co., Ltd., Shanghai, China) in accordance with the manufacturer’s instructions.

### MLST gene amplification, sequencing, and determination

The P scheme of MLST for *B. cereus* group used internal fragments of the following seven housekeeping genes: *glpF* (glycerol uptake facilitator protein), *gmk* (guanylate kinase, putative), *ilvD* (dihydroxy-acid dehydratase), *pta* (phosphate acetyltransferase), *purH* (phosphoribosylaminoimidazolecarboxamide), *pycA* (pyruvate carboxylase) and *tpi* (triosephosphate isomerase)^16^. The seven housekeeping were chosen owing to the following criteria: (1) the common presence in all strain belonged to the core genes; (2) single copy; (3) a relatively high discrimination power relative to rRNA genes and other; (4) no lateral gene transfer and recombination events. PCR amplification of the housekeeping genes was performed using the primer pairs of each gene, different annealing temperatures and extension times (Table S4). The PCR products were purified and subsequently sequenced with the same primers used in the PCR. Each unique gene sequence was regarded as an allele and was assigned an allele number. The set of all allele numbers for a given strain represents an allelic profile or sequence type (ST). Allele numbers of each gene and ST of each strain were assigned using the PubMLST database (http://pubmlst.org/bcereus/). New alleles and new STs were assigned by the MLST website curator.

### Analysis of sequence diversity

Diversity indices of single gene sequence, such as the number of alleles, the number of polymorphic sites, nucleotide diversity per site (*π*), the mean G + C content, the *Ka*/*Ks* ratios (*Ka*: the number of non-synonymous substitutions per non-synonymous site; *Ks*: the number of synonymous substitutions per synonymous site) and Tajima’s D, were analyzed with the software DnaSP version 5.10^29^. Pairwise similarities for the concatenated sequences were analyzed with the software MEGA version 5.05^49^ using Kimura’s two-parameter model^50^.

Allelic linkage disequilibrium was assessed with two test options of both Monte Carlo methods and Parametric (100 resamplings) using LIAN version 3.7 (http://guanine.evolbio.mpg.de/cgi-bin/lian/lian.cgi.pl/query)^25^.

### Population structure

Strain relationships were analyzed using the goeBURST algorithm^26^, as implemented in the software PHYLOViZ^51^ to cluster the STs into clonal complexes (CCs) based on the most stringent definition. The isolates that shared identical alleles at six of the seven loci with at least one other member of the group, were assigned to a single CC. The primary founder of a CC, single locus variants, double locus variants, and singletons were defined using the stringent default setting. Isolate-specific metadata, including the isolation sample and source, were then overlaid on top of the minimum spanning tree.

The population structure of bacteria within the *B. cereus* group was built using the Structure software. The data set was analyzed by the software Structure version 2.3.4 using the admixture model^27^, following a burn-in period of 100,000 iterations, Markov chain Monte Carlo of 50,000 repetitions with five iterations for each K (K set between 2 and 20), where K is the estimated maximum number of genetically distinct groups. The evaluation of the K probability was conducted by the Δ(K) method^52^.

### Phylogenetic analyses

The determined single gene and concatenated sequences (in the following order: *glpF*, *gmk*, *ilvD*, *pta*, *purH*, *pycA* and *tpi*) were aligned using the ClustalW option implemented in the software MEGA version 5.05^29^. The phylogenetic tree of the concatenated genes was constructed using the neighbor-joining (NJ) algorithm^28^ by Kimura’s two-parameter model^50^ with the software MEGA version 5.05^29^. Bootstrap values for individual nodes were calculated for 1,000 replicates for the evaluation of tree robustness.

### Correlation analyses between dDDH, MLST similarities and ANI values

The genomic sequences of 21 representative strains selected on the basis of the phylogenetic analysis were determined by Tianjin Biochip Corporation (Tianjin, China) using the Illumina/Solexa sequencing technology. Reads were trimmed to remove low quality nucleotides using the software Trimmomatic version 0.32 (http://www.usadellab.org/cms/?page=trimmomatic). The high-quality reads of each strain were assembled using the software SOAPdenovo version 1.05. The sequencing depth for each strain was over 100×. Automatic gene annotation for each genome was carried out by the NCBI Prokaryotic Genomes Automatic Annotation Pipeline (PGAAP) (http://www.ncbi.nlm.nih.gov/genomes/static/Pipeline.html), followed by manual editing. The genomic sequences of 11 type strains within the group were obtained from the GenBank database. The dDDH values of 32 bacteria were estimated using the genome-to-genome distance calculator website service (GGDC 2.1) (http://ggdc.dsmz.de/distcalc2.php). ANI values of genomic sequences of them were calculated using the EzGenome web service (http://www.ezbiocloud.net/ezgenome/ani). Correlation analyses between the dDDH values and MLST similarities, dDDH and ANI values were, respectively, simulated using a nonlinear simulation analysis method with the default option of the Curve Fitting Tool implemented in MATLAB 8.1.

### Nucleotide sequence accession numbers

Seven housekeeping genes sequences for 71 strains and draft genomes sequences for 21 strains were submitted to the GenBank database, and all accession numbers were, respectively, shown in Supplementary Tables S2 and S5.

## Electronic supplementary material

Table S1–S5 and Figure S1
