# Supplementary material for: Genetic diversity and population structure of the Bacillus cereus group bacteria from diverse marine environments
Source: Sci Rep. 2017 Apr 6;7:689. doi: 10.1038/s41598-017-00817-1 (PMC5429728; doi:10.1038/s41598-017-00817-1)
Supplement: Supplementary file 1 — Table S1–S5 and Figure S1 [file 41598_2017_817_MOESM1_ESM.pdf]

Genetic diversity and population structure of the *Bacillus cereus* group bacteria from diverse marine environments

Yang Liu[1], Qiliang Lai[1], Juan Du[1], Zongze Shao[1,*]

[1]State Key Laboratory Breeding Base of Marine Genetic Resources; Key Laboratory of Marine Genetic Resources, Third Institute of Oceanography, SOA; Collaborative Innovation Center for Exploitation and Utilization of Marine Biological Resources; Key Laboratory of Marine Genetic Resources of Fujian Province, Xiamen 361005, China

*Corresponding author: Zongze Shao, E-mail: shaozz@163.com.

Supplementary Data

Table S1 Allele profiles and sequence types of 82 strains used in this study

NO.	*glp*	*gmk*	*ilvD*	*pta*	*purH*	*pycA*	*tpi*	STs
BC01	19	2	59	65	19	3	55	761
BC02	19	2	59	65	19	3	55	761
BC03	19	2	59	65	19	3	55	761
BC04	19	2	59	65	19	3	55	761
BC05	19	2	59	65	19	3	55	761
BC06	19	2	59	65	19	3	55	761
BC07	19	2	59	65	19	3	55	761
BC08	19	2	59	65	19	3	55	761
BC09	13	47	9	11	68	12	10	177
BC10	178	27	209	184	131	169	100	764
BC11	13	47	9	11	68	12	10	177
BC12	15	6	29	8	4	7	14	191
BC13	194	115	222	200	190	170	155	765
BC14	19	2	59	65	19	3	55	761
BC15	19	2	59	65	19	3	55	761
BC16	13	47	9	11	68	12	10	177
BC17	19	2	59	65	19	3	55	761
BC18	19	2	59	65	19	3	55	761
BC19	19	2	59	65	19	3	55	761
BC20	13	8	9	201	9	12	23	766
BC21	19	2	59	65	19	3	55	761
BC22	19	2	59	65	19	3	55	761
BC23	13	47	9	11	68	12	10	177
BC24	102	35	107	22	96	20	156	767
BC25	202	116	223	202	118	171	128	769
BC26	15	6	29	46	4	8	21	760
BC27	196	2	224	203	19	3	38	770
BC28	13	47	9	11	68	12	10	177
BC29	33	8	13	19	2	17	17	34
BC30	197	117	225	204	191	172	100	771
BC31	3	23	132	152	38	37	157	772
BC32	24	12	33	205	44	31	158	773
BC33	12	8	43	28	9	12	23	758
BC34	198	22	33	37	192	38	5	774
BC35	199	31	231	206	45	53	159	775
BC36	56	1	93	1	193	37	160	778
BC37	41	6	23	9	4	173	21	779
BC38	13	47	9	11	68	12	10	177
BC39	200	118	226	207	194	174	162	780

BC40	203	115	227	208	195	175	163	781
BC41	72	42	69	42	88	41	30	762
BC42	16	119	170	9	4	7	21	782
BC43	126	120	228	209	4	114	37	783
BC44	201	36	229	210	196	176	32	784
BC45	5	4	3	4	15	6	16	32
BC46	13	47	9	11	68	12	10	177
BC47	13	47	9	11	68	12	10	177
BC48	13	47	9	11	68	12	10	177
BC49	5	4	3	4	15	6	16	32
BC50	5	4	3	4	15	6	16	32
BC51	5	4	3	4	15	6	16	32
BC52	5	4	3	4	15	6	16	32
BC53	5	4	3	4	15	6	16	32
BC54	5	4	3	4	15	6	16	32
BC55	5	4	3	4	15	6	16	32
BC56	19	2	59	65	19	3	55	761
BC57	19	2	59	65	19	3	55	761
BC58	19	2	59	65	19	3	55	761
BC59	194	115	222	200	190	170	155	765
BC60	194	115	222	200	190	170	155	765
BC61	194	115	222	200	190	170	155	765
BC62	194	115	222	200	190	170	155	765
BC63	5	4	3	4	15	6	16	32
BC64	13	47	9	11	68	12	7	759
BC65	5	4	3	4	15	6	16	32
BC66	5	4	3	4	15	6	16	32
BC67	5	4	3	4	15	6	16	32
BC68	5	4	3	4	15	6	16	32
BC69	5	4	3	4	15	6	16	32
BC70	13	47	9	11	68	12	10	177
BC71	13	47	9	11	68	12	10	177
BC72	1	1	1	1	1	1	1	1
BC73	13	125	8	11	11	12	169	921
BC74	211	127	221	221	57	10	171	930
BC75	25	10	22	53	57	23	44	116
BC76	63	13	58	23	25	44	35	83
BC77	15	6	10	8	3	7	14	10
BC78	64	10	9	36	56	22	11	115
BC79	43	26	35	42	39	41	30	111
BC80	195	121	230	211	197	177	161	768
BC81	205	123	107	213	199	10	165	907
BC82	193	114	221	199	189	168	154	763

Table S2 The accession numbers of the genome sequences for 32 strains of the *B. cereus* group

No.	MCCC No./Names	Accession numbers	Source
BC10	MCCC 1A00359	MACI00000000	This study
BC12	MCCC 1A00361	LZRC00000000	This study
BC13	MCCC 1A00365	MACH00000000	This study
BC15	MCCC 1A00395	MACE00000000	This study
BC20	MCCC 1A00432	LZQZ00000000	This study
BC24	MCCC 1A00594	MAOB00000000	This study
BC25	MCCC 1A00732	MAOC00000000	This study
BC26	MCCC 1A00841	LZRA00000000	This study
BC27	MCCC 1A01056	MAOH00000000	This study
BC29	MCCC 1A01404	LZRB00000000	This study
BC30	MCCC 1A01406	MACG00000000	This study
BC31	MCCC 1A01412	LZQW00000000	This study
BC32	MCCC 1A01414	MAOD00000000	This study
BC33	MCCC 1A01874	LZQY00000000	This study
BC34	MCCC 1A02143	MAOF00000000	This study
BC35	MCCC 1A02146	MAOE00000000	This study
BC36	MCCC 1A02161	LZQX00000000	This study
BC39	MCCC 1A04098	MAOI00000000	This study
BC40	MCCC 1A05675	MAOG00000000	This study
BC44	MCCC 1A05942	MACF00000000	This study
BC45	MCCC 1A06182	MACD00000000	This study
BC72	*B. anthracis* ATCC 14578^{T}	ABJC01000000	This study
BC73	*B. cereus* ATCC 14579^{T}	NC_004722.1	The GenBank database
BC74	*B. cytotoxicus* NVH 391-98^{T}	NC_009674.1	The GenBank database
BC75	*B. mycoides* DSM 2048^{T}	NZ_CM000742.1	The GenBank database
BC76	*B. pseudomycoides* DSM 12442^{T}	NZ_CM000745.1	The GenBank database
BC77	*B. thuringiensis* ATCC 10792^{T}	NZ_CM000753.1	The GenBank database
BC78	*B. weihenstephanensis* DSM 11821^{T}	BAUY00000000.1	The GenBank database
BC79	*B. toyonensis* BCT-7112^{T}	NC_022781.1	The GenBank database
BC80	"*B. gaemokensis*" BL3-6^{T}	JOTM00000000	The GenBank database
BC81	"*B. manliponensis*" BL4-6^{T}	JOTN00000000	The GenBank database
BC82	"*B. bingmayongensis*" FJAT-13831^{T}	AKCS01000000	The GenBank database

Table S3 The pairwise dDDH and ANI values among 32 strains within the *B. cereus* group

	BC10	BC12	BC13	BC15	BC20	BC24	BC25	BC26	BC27	BC29	BC30	BC31	BC32	BC33	BC34	BC35	BC36	BC39	BC40	BC44	BC45	BC72	BC73	BC74	BC75	BC76	BC77	BC78	BC79	BC80	BC81	BC82
BC10		95.7	95.2	95.7	95.7	95.8	94.9	95.8	95.7	95.5	95.7	94.9	95.8	95.8	95.9	95.5	95.2	94.8	94.8	95.9	95.8	95.6	95.8	87.9	94.7	90.5	95.7	95.0	94.6	89.9	82.9	89.0
BC12	43.4		94.9	95.4	98.4	94.9	94.7	99.7	95.5	98.7	95.7	94.3	95.7	98.7	95.6	94.9	94.4	94.9	95.0	94.8	95.7	95.1	98.4	88.1	94.1	90.7	99.8	94.3	96.0	90.0	83.6	88.9
BC13	40.7	39.5		94.7	94.4	95.7	97.5	94.9	94.8	94.5	95.2	95.1	95.0	94.5	94.8	95.7	95.1	95.6	99.4	95.8	95.1	94.8	94.5	88.0	96.3	90.6	94.8	96.4	95.1	90.2	83.4	88.9
BC15	44.6	44.4	39.1		95.2	96.3	94.9	95.3	99.2	95.3	97.7	97.4	98.3	95.3	98.6	96.5	97.8	94.8	94.8	96.0	98.2	98.4	95.3	87.7	94.1	89.9	95.3	94.1	94.5	89.4	83.5	88.8
BC20	43.3	72.5	39.3	44.9		95.1	94.6	98.3	95.4	98.9	95.5	94.2	95.5	99.6	95.5	94.8	94.4	94.5	94.6	94.7	95.7	95.0	99.8	88.4	93.9	90.3	98.4	94.1	96.1	90.1	83.5	88.9
BC24	45.2	44.3	40.8	51.1	44.1		95.7	94.8	96.2	94.9	96.2	96.4	96.5	95.1	96.4	97.8	96.6	94.9	95.7	97.8	96.5	96.1	95.2	88.3	94.9	90.1	94.8	95.3	94.8	89.7	83.2	88.5
BC25	40.0	39.3	58.4	39.7	39.0	42.1		94.7	95.1	94.5	95.3	95.0	95.2	94.6	95.0	95.9	95.2	95.7	97.6	95.9	95.4	95.1	94.7	88.4	95.7	90.8	94.6	95.9	94.8	90.1	83.5	89.1
BC26	43.2	82.7	39.5	44.3	70.9	44.1	39.3		95.4	98.6	95.7	94.3	95.6	98.7	95.6	94.8	94.5	94.8	95.0	94.8	95.5	95.2	98.4	88.1	94.2	90.7	99.5	94.3	96.0	90.0	83.6	89.0
BC27	44.3	44.4	39.2	78.4	44.9	51.0	39.7	44.3		95.4	98.1	97.6	98.4	95.4	98.6	96.7	97.3	94.9	94.9	96.1	98.2	97.9	95.4	87.7	94.1	90.0	95.4	94.2	94.7	89.5	83.5	88.6
BC29	43.4	70.2	39.4	45.1	82.3	44.1	39.1	70.0	44.8		95.6	94.2	95.6	99.2	95.5	94.8	94.3	94.4	94.6	94.7	95.5	95.0	98.8	87.9	94.0	90.3	98.6	94.1	95.9	89.9	83.3	88.6
BC30	45.3	45.2	39.8	61.9	45.4	51.8	39.9	44.9	62.4	45.4		96.7	98.5	95.5	98.5	96.7	96.7	94.9	95.3	96.1	97.5	97.0	95.5	87.6	94.5	90.5	95.5	94.6	94.8	89.7	83.4	88.9
BC31	44.2	44.2	39.2	64.3	44.5	51.4	39.6	43.7	63.6	44.6	59.6		96.9	94.2	96.8	96.7	98.8	94.6	95.2	96.3	97.2	98.1	94.3	88.2	94.4	90.1	94.2	94.6	93.9	89.6	83.2	88.5
BC32	45.1	45.5	39.7	65.9	45.9	52.0	39.9	45.2	65.9	45.7	72.9	62.2		95.6	99.5	96.7	96.9	94.7	95.1	96.2	97.7	97.6	95.5	87.7	94.2	90.2	95.5	94.3	94.7	89.4	83.3	88.7
BC33	43.3	70.8	39.2	45.5	86.4	44.2	37.7	70.1	44.8	80.7	45.6	44.5	45.7		95.5	94.8	94.5	94.5	94.6	94.8	95.7	95.1	99.4	88.2	94.0	90.4	98.7	94.2	96.0	90.1	83.5	89.0
BC34	44.9	45.4	39.6	62.5	45.8	52.1	57.5	45.0	63.7	45.8	71.2	60.4	79.9	45.6		96.6	96.9	94.8	94.9	96.1	97.6	97.6	95.5	87.6	94.1	90.1	95.5	94.2	94.6	89.4	83.3	88.7
BC35	44.1	43.5	39.1	52.7	43.5	54.2	40.1	43.6	52.1	43.8	52.2	52.5	53.0	43.6	53.2		96.9	94.7	95.7	97.7	96.9	96.3	94.9	88.2	94.7	90.1	94.8	94.9	94.8	90.1	83.8	88.6
BC36	44.0	44.2	39.3	61.9	44.7	51.0	39.7	44.0	61.3	44.7	58.4	73.4	59.6	44.3	58.1	52.1		94.8	95.1	96.4	97.2	98.9	94.5	87.8	94.6	89.8	94.3	94.6	93.9	89.5	83.4	88.2
BC39	38.0	37.2	43.8	36.6	37.0	37.6	43.7	37.1	36.7	37.1	37.0	36.9	36.9	37.0	36.9	36.6	36.8		95.6	94.8	95.0	94.6	94.6	88.5	96.1	90.8	94.8	96.1	94.3	90.3	83.4	89.2
BC40	40.5	39.5	87.1	39.2	39.3	40.9	57.9	39.6	39.2	39.4	39.7	39.1	39.6	39.3	39.5	39.1	39.2	44.0		95.5	95.2	94.9	94.7	88.1	95.8	90.6	94.9	95.9	95.4	90.1	83.4	88.8
BC44	44.1	42.7	39.9	49.9	43.2	57.9	41.3	42.9	51.5	43.0	50.7	49.6	50.6	43.1	51.2	54.4	50.0	37.3	40.0		96.4	96.0	94.8	88.3	95.2	90.3	94.7	95.4	94.6	89.9	83.1	88.5
BC45	44.3	44.0	39.2	65.4	44.4	51.5	39.9	44.0	64.8	44.5	60.0	57.3	60.7	44.7	60.2	52.4	56.5	36.7	39.2	51.7		97.6	95.8	87.9	94.3	89.9	95.5	94.5	94.8	89.5	83.6	88.7
BC72	43.9	43.9	39.0	61.4	44.1	50.8	39.5	43.8	60.7	44.4	58.7	73.9	60.2	44.1	58.2	52.2	79.9	36.6	39.0	49.1	55.6		95.1	87.5	94.4	89.8	95.0	94.5	94.2	89.3	83.0	88.3
BC73	43.4	71.7	39.4	45.2	89.2	44.1	39.1	70.9	45.0	82.3	45.7	44.7	46.2	85.4	45.8	43.9	44.7	37.3	39.5	43.0	44.4	44.6		88.5	94.0	90.3	98.4	94.1	96.3	90.0	83.5	89.0
BC74	25.5	25.3	25.6	25.4	25.5	25.5	25.6	25.4	25.5	25.5	25.5	25.4	25.5	25.4	25.5	25.4	25.6	25.9	25.7	25.5	25.6	25.5	26.0		88.2	89.7	88.2	88.3	88.1	88.6	83.1	87.3
BC75	38.9	38.3	50.2	38.1	38.0	40.5	53.3	38.3	38.0	38.1	38.3	38.0	38.3	38.1	38.4	38.6	38.1	45.6	50.1	39.1	38.2	38.0	38.1	25.9		90.8	93.9	99.2	93.8	90.3	83.0	89.3
BC76	26.9	27.0	28.1	26.8	26.9	27.0	27.7	27.0	26.8	26.9	26.9	26.8	26.7	27.0	26.8	26.9	26.8	28.1	28.2	26.7	26.7	26.9	27.1	28.0	28.0		90.8	90.9	90.1	92.9	84.7	92.0
BC77	43.3	86.7	39.5	44.5	71.0	44.1	39.3	81.2	44.2	70.2	45.0	43.9	45.2	71.1	45.1	43.1	43.9	37.1	39.4	42.8	43.7	43.7	71.2	25.5	38.3	27.0		94.1	96.0	90.1	83.7	89.0
BC78	38.9	38.0	49.3	38.2	37.9	40.5	52.7	38.1	38.1	38.1	38.5	38.0	38.3	37.9	38.3	38.5	38.1	45.5	49.2	39.4	38.1	37.9	37.9	25.7	78.2	27.9	38.1		94.0	90.5	83.4	89.5
BC79	43.6	44.8	42.4	42.8	44.8	44.0	42.0	44.8	42.6	44.6	43.2	42.7	43.3	44.7	43.2	42.6	42.7	39.3	42.3	42.7	42.5	42.8	45.0	26.2	41.2	27.3	45.0	41.1		89.9	83.3	88.3
BC80	26.3	26.2	27.0	26.3	26.3	26.3	27.0	26.2	26.2	26.3	26.3	26.3	26.4	26.3	26.4	26.4	26.1	27.4	27.1	26.3	26.3	26.2	26.4	27.3	27.1	34.0	26.2	27.1	26.7		84.1	90.7
BC81	22.2	22.2	22.5	22.2	22.4	22.4	22.4	22.2	22.3	22.3	22.4	22.2	22.3	22.3	22.2	22.1	22.2	22.5	22.4	22.4	22.4	22.2	22.7	23.0	22.5	23.5	22.3	22.4	22.7	23.0		82.4
BC82	26.7	27.0	27.6	26.7	27.0	26.8	27.4	26.9	26.7	26.9	26.7	26.8	26.7	27.1	26.8	26.9	26.7	27.6	27.7	26.5	26.8	26.7	27.4	27.8	27.4	42.0	27.1	27.5	27.3	34.5	23.6	

Note: The dDDH and ANI values were respectively shown in the lower-left corner and upper-right corner of Table S3.

Table S4 The primer pairs of seven shousekeeping genes in *B. cereus* group MLST

Name	Forward primer 5'→3' sequence	Annealing temperature (℃)	Extension time (s)
glpF-F	GCGTTTGTGCTGGTGTAAGT	59	30
glpF-R	CTGCAATCGGAAGGAAGAAG		
gmk-F	ATTTAAGTGAGGAAGGGTAGG	56	40
gmk-R	GCAATGTTCACCAACCACAA		
ilvD-F	CGGGGCAAACATTAAGAGAA	58	30
ilvD-R	GGTTCTGGTCGTTTCCATTC		
pta-F	GCAGAGCGTTTAGCAAAAGAA	56	30
pta-R	TGCAATGCGAGTTGCTTCTA		
purH-F	CTGCTGCGAAAAATCACAAA	56	30
purH-R	CTCACGATTCGCTGCAATAA		
pycA-F	GCGTTAGGTGGAAACGAAAG	57	30
pycA-R	CGCGTCCAAGTTTATGGAAT		
tpi-F	GCCCAGTAGCACTTAGCGAC	58	30
tpi-R	CCGAAACCGTCAAGAATGAT		

The data of the primer pairs were from the P scheme for *B. cereus* group MLST.

The capital “F” and “R” in the name of primer pairs represented respectively a forward primer and a reverse primer.

Table S5 The accession numbers of seven housekeeping genes for 71 strains used in this study.

NO.	*glpF*	*gmk*	*ilvD*	*pta*	*purH*	*pycA*	*tpi*
BC01	KJ812761	KJ812548	KJ812477	KJ812690	KJ812903	KJ812619	KJ812832
BC02	KJ812762	KJ812549	KJ812478	KJ812691	KJ812904	KJ812620	KJ812833
BC03	KJ812763	KJ812550	KJ812479	KJ812692	KJ812905	KJ812621	KJ812834
BC04	KJ812764	KJ812551	KJ812480	KJ812693	KJ812906	KJ812622	KJ812835
BC05	KJ812765	KJ812552	KJ812481	KJ812694	KJ812907	KJ812623	KJ812836
BC06	KJ812766	KJ812553	KJ812482	KJ812695	KJ812908	KJ812624	KJ812837
BC07	KJ812767	KJ812554	KJ812483	KJ812696	KJ812909	KJ812625	KJ812838
BC08	KJ812768	KJ812555	KJ812484	KJ812697	KJ812910	KJ812626	KJ812839
BC09	KJ812769	KJ812556	KJ812485	KJ812698	KJ812911	KJ812627	KJ812840
BC10	KJ812770	KJ812557	KJ812486	KJ812699	KJ812912	KJ812628	KJ812841
BC11	KJ812771	KJ812558	KJ812487	KJ812700	KJ812913	KJ812629	KJ812842
BC12	KJ812772	KJ812559	KJ812488	KJ812701	KJ812914	KJ812630	KJ812843
BC13	KJ812773	KJ812560	KJ812489	KJ812702	KJ812915	KJ812631	KJ812844
BC14	KJ812774	KJ812561	KJ812490	KJ812703	KJ812916	KJ812632	KJ812845
BC15	KJ812775	KJ812562	KJ812491	KJ812704	KJ812917	KJ812633	KJ812846
BC16	KJ812776	KJ812563	KJ812492	KJ812705	KJ812918	KJ812634	KJ812847
BC17	KJ812777	KJ812564	KJ812493	KJ812706	KJ812919	KJ812635	KJ812848
BC18	KJ812778	KJ812565	KJ812494	KJ812707	KJ812920	KJ812636	KJ812849
BC19	KJ812779	KJ812566	KJ812495	KJ812708	KJ812921	KJ812637	KJ812850
BC20	KJ812780	KJ812567	KJ812496	KJ812709	KJ812922	KJ812638	KJ812851
BC21	KJ812781	KJ812568	KJ812497	KJ812710	KJ812923	KJ812639	KJ812852
BC22	KJ812782	KJ812569	KJ812498	KJ812711	KJ812924	KJ812640	KJ812853
BC23	KJ812783	KJ812570	KJ812499	KJ812712	KJ812925	KJ812641	KJ812854
BC24	KJ812784	KJ812571	KJ812500	KJ812713	KJ812926	KJ812642	KJ812855
BC25	KJ812785	KJ812572	KJ812501	KJ812714	KJ812927	KJ812643	KJ812856
BC26	KJ812786	KJ812573	KJ812502	KJ812715	KJ812928	KJ812644	KJ812857
BC27	KJ812787	KJ812574	KJ812503	KJ812716	KJ812929	KJ812645	KJ812858
BC28	KJ812788	KJ812575	KJ812504	KJ812717	KJ812930	KJ812646	KJ812859
BC29	KJ812789	KJ812576	KJ812505	KJ812718	KJ812931	KJ812647	KJ812860
BC30	KJ812790	KJ812577	KJ812506	KJ812719	KJ812932	KJ812648	KJ812861
BC31	KJ812791	KJ812578	KJ812507	KJ812720	KJ812933	KJ812649	KJ812862
BC32	KJ812792	KJ812579	KJ812508	KJ812721	KJ812934	KJ812650	KJ812863
BC33	KJ812793	KJ812580	KJ812509	KJ812722	KJ812935	KJ812651	KJ812864
BC34	KJ812794	KJ812581	KJ812510	KJ812723	KJ812936	KJ812652	KJ812865
BC35	KJ812795	KJ812582	KJ812511	KJ812724	KJ812937	KJ812653	KJ812866
BC36	KJ812796	KJ812583	KJ812512	KJ812725	KJ812938	KJ812654	KJ812867
BC37	KJ812797	KJ812584	KJ812513	KJ812726	KJ812939	KJ812655	KJ812868
BC38	KJ812798	KJ812585	KJ812514	KJ812727	KJ812940	KJ812656	KJ812869
BC39	KJ812799	KJ812586	KJ812515	KJ812728	KJ812941	KJ812657	KJ812870
BC40	KJ812800	KJ812587	KJ812516	KJ812729	KJ812942	KJ812658	KJ812871
BC41	KJ812801	KJ812588	KJ812517	KJ812730	KJ812943	KJ812659	KJ812872
BC42	KJ812802	KJ812589	KJ812518	KJ812731	KJ812944	KJ812660	KJ812873
BC43	KJ812803	KJ812590	KJ812519	KJ812732	KJ812945	KJ812661	KJ812874

BC44	KJ812804	KJ812591	KJ812520	KJ812733	KJ812946	KJ812662	KJ812875
BC45	KJ812805	KJ812592	KJ812521	KJ812734	KJ812947	KJ812663	KJ812876
BC46	KJ812806	KJ812593	KJ812522	KJ812735	KJ812948	KJ812664	KJ812877
BC47	KJ812807	KJ812594	KJ812523	KJ812736	KJ812949	KJ812665	KJ812878
BC48	KJ812808	KJ812595	KJ812524	KJ812737	KJ812950	KJ812666	KJ812879
BC49	KJ812809	KJ812596	KJ812525	KJ812738	KJ812951	KJ812667	KJ812880
BC50	KJ812810	KJ812597	KJ812526	KJ812739	KJ812952	KJ812668	KJ812881
BC51	KJ812811	KJ812598	KJ812527	KJ812740	KJ812953	KJ812669	KJ812882
BC52	KJ812812	KJ812599	KJ812528	KJ812741	KJ812954	KJ812670	KJ812883
BC53	KJ812813	KJ812600	KJ812529	KJ812742	KJ812955	KJ812671	KJ812884
BC54	KJ812814	KJ812601	KJ812530	KJ812743	KJ812956	KJ812672	KJ812885
BC55	KJ812815	KJ812602	KJ812531	KJ812744	KJ812957	KJ812673	KJ812886
BC56	KJ812816	KJ812603	KJ812532	KJ812745	KJ812958	KJ812674	KJ812887
BC57	KJ812817	KJ812604	KJ812533	KJ812746	KJ812959	KJ812675	KJ812888
BC58	KJ812818	KJ812605	KJ812534	KJ812747	KJ812960	KJ812676	KJ812889
BC59	KJ812819	KJ812606	KJ812535	KJ812748	KJ812961	KJ812677	KJ812890
BC60	KJ812820	KJ812607	KJ812536	KJ812749	KJ812962	KJ812678	KJ812891
BC61	KJ812821	KJ812608	KJ812537	KJ812750	KJ812963	KJ812679	KJ812892
BC62	KJ812822	KJ812609	KJ812538	KJ812751	KJ812964	KJ812680	KJ812893
BC63	KJ812823	KJ812610	KJ812539	KJ812752	KJ812965	KJ812681	KJ812894
BC64	KJ812824	KJ812611	KJ812540	KJ812753	KJ812966	KJ812682	KJ812895
BC65	KJ812825	KJ812612	KJ812541	KJ812754	KJ812967	KJ812683	KJ812896
BC66	KJ812826	KJ812613	KJ812542	KJ812755	KJ812968	KJ812684	KJ812897
BC67	KJ812827	KJ812614	KJ812543	KJ812756	KJ812969	KJ812685	KJ812898
BC68	KJ812828	KJ812615	KJ812544	KJ812757	KJ812970	KJ812686	KJ812899
BC69	KJ812829	KJ812616	KJ812545	KJ812758	KJ812971	KJ812687	KJ812900
BC70	KJ812830	KJ812617	KJ812546	KJ812759	KJ812972	KJ812688	KJ812901
BC71	KJ812831	KJ812618	KJ812547	KJ812760	KJ812973	KJ812689	KJ812902

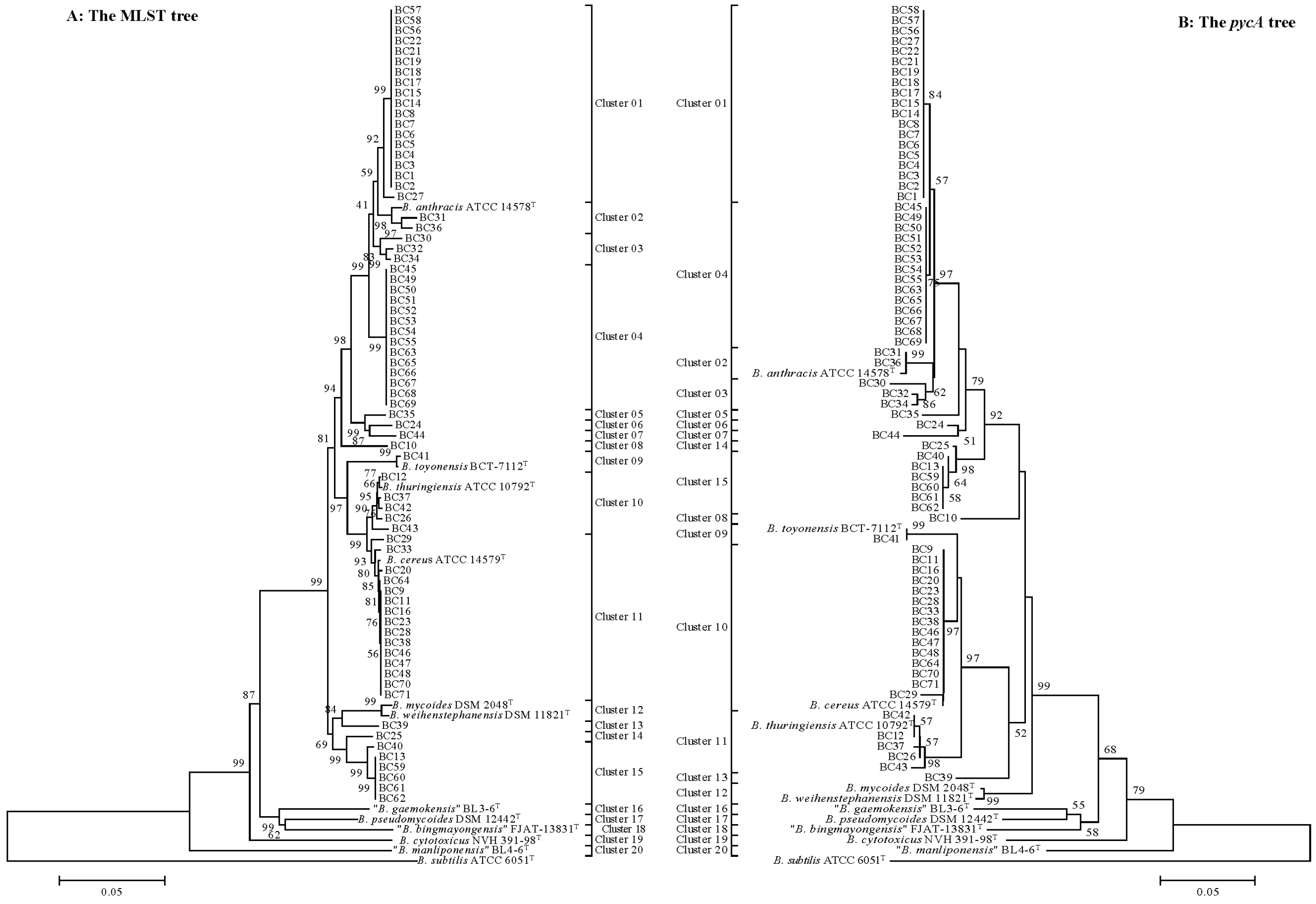

Figure S1 The comparison of phylogenetic trees based on, respectively, seven concatenated housekeeping genes and *pycA* gene sequences
